# Ischemic postconditioning protects against acute kidney injury after limb ischemia reperfusion by regulating HMGB1 release and autophagy

**DOI:** 10.1080/0886022X.2023.2189482

**Published:** 2023-05-09

**Authors:** Zhongdi Liu, Yifan Chen, Zhe Du, Fengxue Zhu, Wei Huang

**Affiliations:** National Center for Trauma Medicine, Ministry of Education Key Laboratory of Trauma and Neural Regeneration, Trauma Medicine Center, Peking University People’s Hospital, Beijing, China

**Keywords:** Limb ischemia, ischemic postconditioning, kidney, autophagy, HMGB1

## Abstract

Ischemic postconditioning (I-PostC) has a protective effect against acute kidney injury (AKI) induced by limb ischemia–reperfusion (LIR); however, the exact mechanism remains to be elucidated. Our study aims to investigate the potential involvement of high-mobility group box 1 protein (HMGB1) and autophagy in renoprotection generated by I-PostC. A rat model of LIR-induced AKI was established and rats were randomly assigned to five groups: (i) sham-operated control, (ii) I/R, (iii) I/R + I-PostC, (iv) I/R + I-PostC + rapamycin (autophagy activator), and (v) I/R + I-PostC + 3-methyladenine (autophagy inhibitor). Morphological changes in the kidneys were assessed by histology, and ultrastructural changes in renal tubular epithelial cells and glomerular podocytes were observed by transmission electron microscopy. The levels of kidney function parameters, serum inflammatory factors, and autophagy markers were detected. The results showed that the levels of HMGB1, Beclin1, LC3-II/LC3-I, and inflammatory cytokines (TNF-α and IL-6) were significantly higher in the I/R group compared to the sham control in serum and in renal tissues. I-PostC significantly reduced the levels of HMGB1, Beclin1, LC3-II/LC3-I, and inflammatory cytokines in renal tissues and improved renal function. Renal histopathology and ultrastructural observations indicated that I-PostC alleviated renal tissue injury. In addition, rapamycin (autophagy activator) treatment increased the levels of inflammatory cytokine expression levels and decreased renal function, reversed the protective effect of I-PostC against LIR-induced AKI. In conclusion, I-PostC could play a protective role against AKI by regulating the release of HMGB1 and inhibiting autophagy activation.

## Introduction

Limb ischemia–reperfusion (LIR) injury after severe trauma often leads to a systemic inflammatory response, resulting in distal organ injury [1–3]. Since the kidney is one of the main target organs, acute kidney injury (AKI) induced by LIR can lead to a rapid loss of renal function and high mortality. At present, this type of disease has attracted worldwide attention; however, the pathophysiological mechanism of LIR-induced AKI remains unclear [4,5].

Previous studies have shown that ischemia–reperfusion (I/R)-induced AKI is associated with inflammatory response activation, excessive oxygen free radical generation, and calcium overload [6,7]. During I/R injury, stimuli, such as acute oxidative stress, lead to the release of endogenous cellular autoantigens, including high-mobility group box 1 protein (HMGB1) and heat shock proteins (HSPs), also known as damage-associated molecular patterns (DAMPs) [8,9]. DAMPs, in turn, activate transcription factors through the mitogen-activated protein kinase (MAPK) and nuclear factor of kappa light polypeptide gene enhancer in B-cells (NF-κB) signaling pathways by binding to corresponding receptors and triggering inflammatory responses [10].

In addition, several studies have reported that autophagy plays a role in the pathogenesis of I/R injury in organs, such as the heart, lungs, and liver [11,12]. Autophagy is an evolutionary conserved metabolic process that occurs in all eukaryotic cells and is responsible for the maintenance of the intracellular homeostasis, as well as other cellular processes, such as the renewal of cytoplasmic components, biological growth, and immune regulation [13]. Sepsis, acute ischemia, and drug- and heavy metal-induced renal toxicity have been shown to activate autophagy [14]. Under physiological conditions, autophagy has a protective effect on cells; however, the overactivation of autophagy may cause cell damage, including cell death. Several studies have described the important mediating role of autophagy in acute and chronic kidney injury [15]. Furthermore, increasing evidence suggests that there exists an interaction between autophagy and inflammation during the pathogenesis of a wide range of human diseases [16,17].

Ischemic postconditioning (I-PostC) is the process of programmed transient I/R, which induces tissue adaptation to prolonged ischemia and reperfusion. Although a large number of animal studies have confirmed that I-PostC could effectively alleviate LIR-induced AKI, the specific molecular mechanism is not fully understood [18,19]. Our previous study demonstrated that I-PostC may protect against LIR-induced AKI by inhibiting the Toll-like receptor 4 (TLR4)/NF‑κB signaling pathway and reducing the expression of proinflammatory cytokines [20]. Current studies have shown that HMGB1 is an important inflammatory promoter and upstream molecule of TLR4/NF‑κB signaling pathway; therefore, HMGB1 could also be involved in the development of AKI after LIR. However, it is not known whether autophagy is involved in the renal protection by I-PostC. Thus, we established a LIR-induced AKI rat model and investigated the role of HMGB1 and autophagy in the renoprotective effect of I-PostC.

## Materials and methods

### Animal preparation and experimental procedures

This study was approved by the Animal Research Ethics Committee of Peking University People’s Hospital (approval number: 2020PHE067). All animal experiments were conducted in accordance with the guidelines of the National Institutes of Health for the Care and Use of Laboratory Animals.

Thirty adult male Sprague-Dawley rats (Vital River Biological Co., Ltd., Beijing, China), aged 8–10 weeks and weighing 300–350 g, were obtained from the Animal Center of Peking University People’s Hospital. All experimental animals were specific pathogen free, housed in separate cages with a stable temperature and relative humidity, and had free access to food and water until 12 h before the experiment.

**Figure 1.**
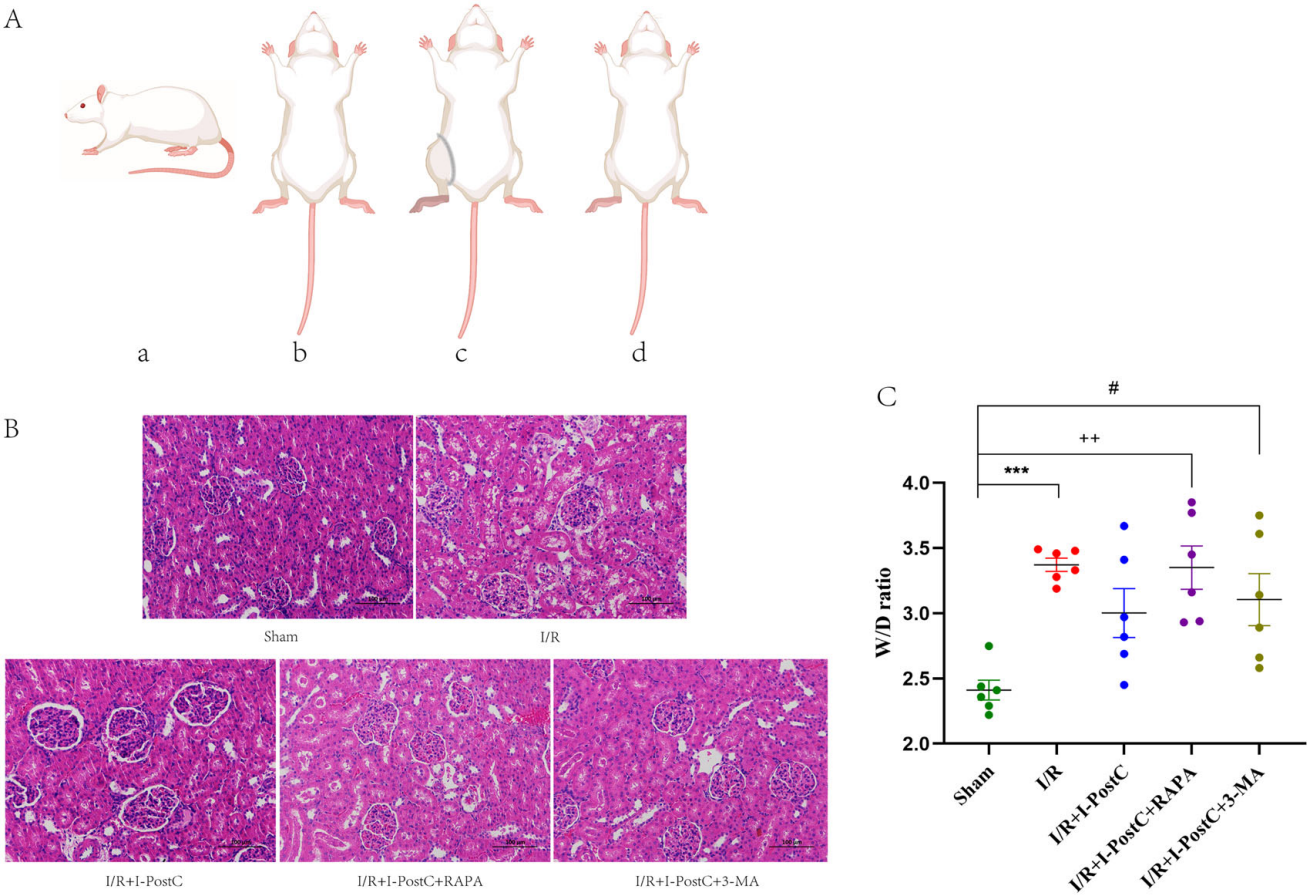
Schematic diagram of the rat model establishment (A). Normal rat (a); rat under anesthesia (b); rat in the state of limb ischemia, the blood flow of the right hind limb was blocked by a self-locking nylon band (width 5 mm) above the trochanter (c); rat in the state of limb ischemia–reperfusion, the nylon band was removed after ischemic intervention (d). Morphological features evaluated by H&E staining (×200) and W/D ratio of the kidneys in different groups (B, C). Sham group, normal glomeruli and tubules; I/R group, segmental renal tubular epithelial edema, lumen narrowing, and local glomerular atrophy; I/R + I-PostC group, mild edema of renal tubular epithelial cells and local lumen stenosis; I/R + I-PostC + RAPA group, extensive glomerular atrophy with mild to moderate tubular stenosis; I/R + I-PostC + 3-MA group, slight edema of tubular epithelial cells. (C) ***p<.001, I/R vs. sham; ^++^*p*<.01, RAPA vs. I/R; ^#^*p*<.05, 3-MA vs. I/R.

The animal model of LIR-induced AKI was established using the same protocol as previously reported [20], the duration and sequence of I-PostC had been widely recognized [21,22]. A self-locking nylon band (5 mm in width) was used to block arterial blood flow in the rat’s right hind limb above the trochanter, when the paw of the rat became pale and cool, and the laser Doppler blood flow imaging verified the occlusion of the blood flow, the limb ischemia model was established successfully. When a certain ischemia time was maintained, the nylon band was cut and the blood supply to the limb was restored, that is, the LIR model (Figure 1(A)). I-PostC in this study refers to the intervention of 5 min limb ischemia and 5 min limb reperfusion for three cycles (30 min in total) between blood flow blocking and blood flow recovery of the right hind limb, which was also accomplished through the use of nylon band. The rats (*n* = 30) were randomly divided into five groups (numbered from 1 to 5, *n* = 6/group): the sham group (group 1, anesthesia procedure only, no limb I/R), the I/R group (group 2, 4 h of reperfusion after 4 h of limb ischemia under anesthesia), the I/R + I-PostC group (group 3, 4 h of ischemia followed by three cycles of 5 min of limb ischemia and 5 min of reperfusion, and then 4 h of reperfusion was conducted), the rapamycin (I/R + I-PostC + RAPA) group (group 4, rats were intraperitoneally (i.p.) injected with autophagy activator rapamycin, 1.0 mg/kg, 30 min before treatment described for group 3), and the 3-methyladenine (I/R + I-PostC + 3-MA) group (group 5, rats were i.p. injected with autophagy inhibitor 3-MA, 15.0 mg/kg, 30 min before treatment described for group 3). Rats in groups 1 and 2 were i.p. injected with the same volume of normal saline.

All rats were anesthetized by Zoletil 50 injection (40 mg/kg, i.p.), and a warming table with constant temperature was used to maintain body temperature.

Additional doses of 10% of the initial dose were administered as required to maintain anesthesia. All rats survived the establishment of the model, blood and kidney tissues were collected under anesthesia after surgery.

### Histological analysis

Rat kidneys were collected and perfused with cold saline and 4% paraformaldehyde. The tissues were fixed at 4 °C for 24 h, embedded in paraffin, cut into 5 μm sections, and stained with hematoxylin and eosin (H&E) at room temperature for 5 min. Kidney sections were then evaluated under a standard light microscope (ECLIPSE E100, Nikon, Tokyo, Japan) by two experienced pathologists blinded to the study through three randomly selected fields. Microscopic evaluation of all sections was performed randomly. The pathological observation of renal injury included the following parameters: the degree of tubular dilatation, casting, vacuolization, and necrosis.

### Analysis of serum and inflammatory factors in kidney tissue

Blood samples were collected from the inferior vena cava and centrifuged at 3000 rpm for 10 min. Each sample was evaluated for blood urea nitrogen (BUN), serum creatinine (SCr), and creatine kinase (CK) using clinically automated analysis methods (Rayto, Chemray 800, Shenzhen, China). The concentrations of HMGB1, tumor necrosis factor-α (TNF‑α), and interleukin-6 (IL‑6) in the serum were measured using ELISA kits according to the manufacturer’s instructions (MultiSciences Biotech Co., Ltd., Hangzhou, China and Thermo Fisher Scientific Inc., Waltham, MA). The concentrations of HMGB1, NF-κB, TNF-α, and IL-6 in kidney tissues were also measured using ELISA kits. The absorbance at 450 nm was measured using a multifunctional microplate reader (BioTek Epoch, San Diego, CA).

### Immunohistochemical analysis

Renal tissues were fixed with 10% neutral buffered formalin for 24 h, embedded in paraffin, and sectioned at 5 μm according to the standard procedure. The sections were deparaffinized, gradually hydrated, microwaved at 100 °C in sodium citrate buffer for 20 min, cooled to room temperature, and then incubated in 3% H_2_O_2_ for 10 min. The sections were blocked using bovine serum albumin at room temperature for 30 min and then incubated at 4 °C overnight with a primary antibody against HMGB1 (1:500; cat. no. GB11103; Servicebio, Woburn, MA) and primary antibody against NF-κB (1:100; cat. no. #38054; Signalway, College Park, MD). Next, samples were washed with phosphate-buffered saline three times for 5 min each wash, and incubated with horseradish peroxidase labeled goat anti-mouse secondary antibody (1:200, cat. no. GB23301; Servicebio, Woburn, MA). The samples were developed using 3,3′-diaminobenzidine (DAB; no. G1211; Servicebio, Woburn, MA) for 30 s at room temperature. After dehydration and drying, the sections were mounted using neutral glue and evaluated using microscope and the Image-Pro Plus software. The protein expression levels of the HMGB1 and NF-κB p65 were determined using the average optical density (AOD) of staining at ×200 magnification. IHC evaluation was evaluated with three randomly selected fields by two experienced pathologists blinded to the study.

### Western blot analysis

Total protein was extracted from the kidney tissues as previously described. Protein samples (50 μg/lane) were separated on a sodium dodecyl sulfate-polyacrylamide gel (12% gel) and transferred onto polyvinylidene fluoride membranes. The membranes were blocked in 5% nonfat milk for 1 h at room temperature and then incubated with primary antibodies on a rotating shaker overnight at 4 °C. The following primary antibodies were used: anti-β-actin (1:1000; cat. no. GB12001; Servicebio, Woburn, MA), anti-HMGB1 (1:2000; cat. no. GB11103; Servicebio, Woburn, MA), anti-Beclin 1 (1:1000; cat. no. GB13228-2; Servicebio, Woburn, MA), and anti-microtubule-associated protein 1 light chain 3, anti-LC3 (1:3000; cat. no. 14600-1-AP; Servicebio, Woburn, MA). Next, the membranes were washed three times with tris-buffered saline containing Tween20 (TBST), and then incubated with a horseradish peroxidase‑conjugated secondary antibody (1:3000; cat. no. GB23302, GB23303; Servicebio, Woburn, MA) at room temperature for 1 h. The membranes were subsequently washed three times with TBST and visualized using electrochemiluminescence. The Image Lab Analysis System (Alpha Innotech, alphaEaseFC, San Leandro, CA; Adobe, Adobe PhotoShop, San Jose, CA) was used to analyze the images.

### Transmission electron microscopy (TEM)

The kidney tissues were collected, cut into 1 mm^3^ blocks, and then fixed in 2.5% glutaraldehyde overnight at 4 °C. The tissue blocks were washed with PBS (pH 7.4), fixed in 1% osmic acid, gradually dehydrated with ethanol and acetone, embedded in epoxy resin, and incubated at 70 °C for 48 h to allow for resin polymerization. Next, the tissues were cut into ultrathin sections, double stained with uranyl acetate and lead citrate, and then examined by using a Hitachi HT7700 TEM (Hitachi, Tokyo, Japan).

### Statistical analysis

All data were presented as the mean ± standard deviation (SD). One-way analysis of variance (ANOVA) with Bonferroni’s *post hoc* test was used to compare multiple groups. The level of statistical significance for all analyses was set at *p*<.05. Analysis was performed using the SPSS version 19.0 software (IBM Corp., Armonk, NY).

## Results

### Limb ischemia–reperfusion injury induced morphological and functional kidney impairment

**Figure 2.**
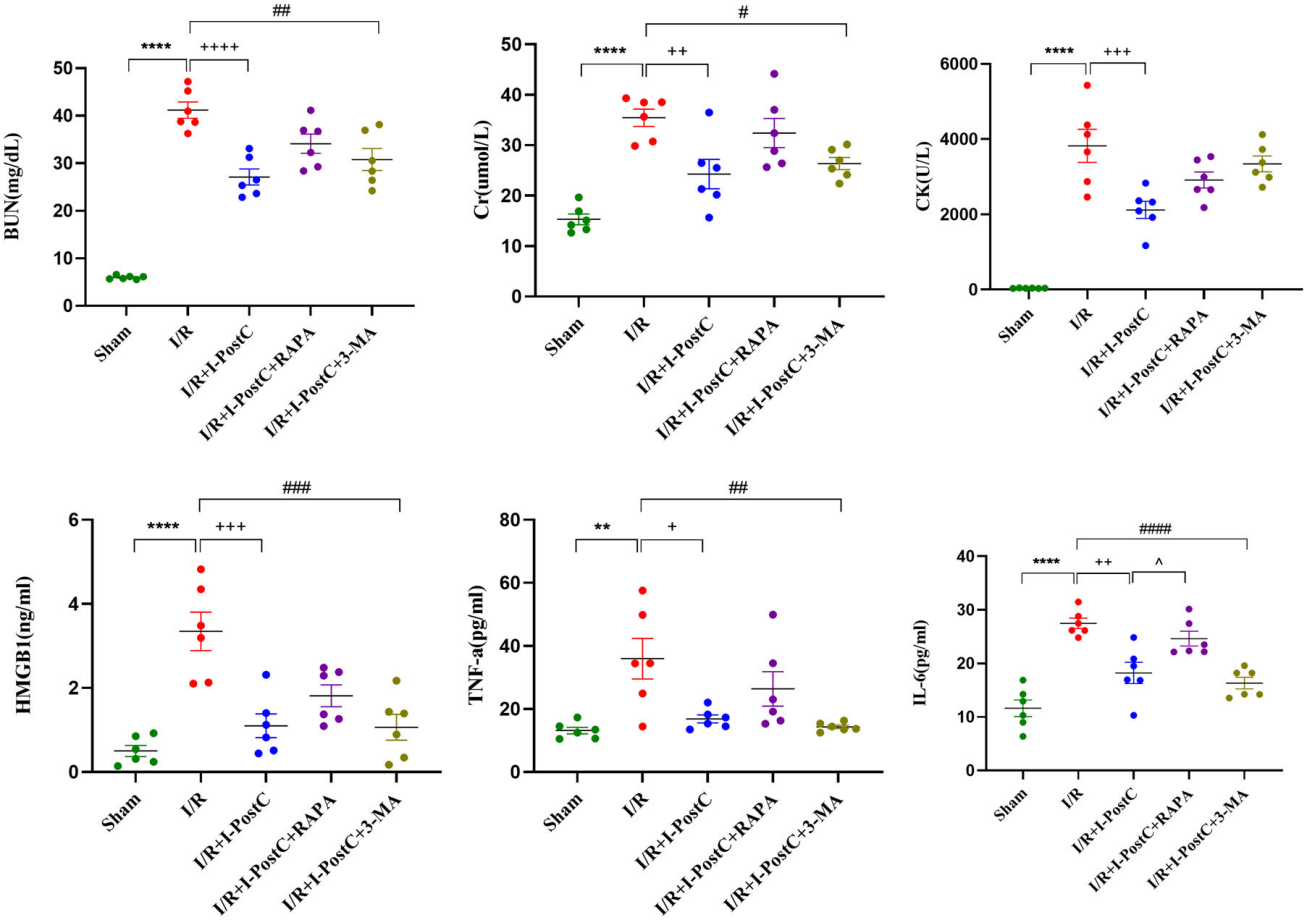
Levels of biochemical parameters (BUN, Cr, and CK) and inflammatory cytokines (HMGB1, TNF-α, and IL-6) in rat serum samples. Data are presented as the mean ± SD, *N* = 6/group. ****p<.0001, **p<.01, I/R vs. sham; ^++++^*p*<.0001, ^+++^*p*<.001, ^++^*p*<.01, ^+^*p*<.05, I/R + I-PostC vs. I/R; ^####^*p*<.0001, ^###^*p*<.001, ^##^*p*<.01, ^#^*p*<.05, I/R + I-PostC + 3-MA vs. I/R; ^∧^*p*<.05, I/R + I-PostC + RAPA vs. I/R + I-PostC. Cr: creatinine; BUN: blood urine nitrogen; CK: creatine kinase.

As shown in Figure 1(B), the renal morphology in the sham group was normal. In the I/R group, in addition to the large number of necrotic renal tubular epithelial cells and edema, uneven lumen stenosis and occlusion were observed. The wet/dry weight ratio of the kidney was significantly higher in rats from the I/R group than in the sham group (Figure 1(C)). Serum analysis showed that the levels of BUN, Cr, and CK were significantly increased in the I/R group compared to the sham group (Figure 2). These results suggested that LIR induced pathological changes in the kidney tissues and impaired the renal function, validating the effective establishment of the LIR-induced AKI model.

### I/R-induced acute kidney injury elevated inflammatory response and autophagy

**Figure 3.**
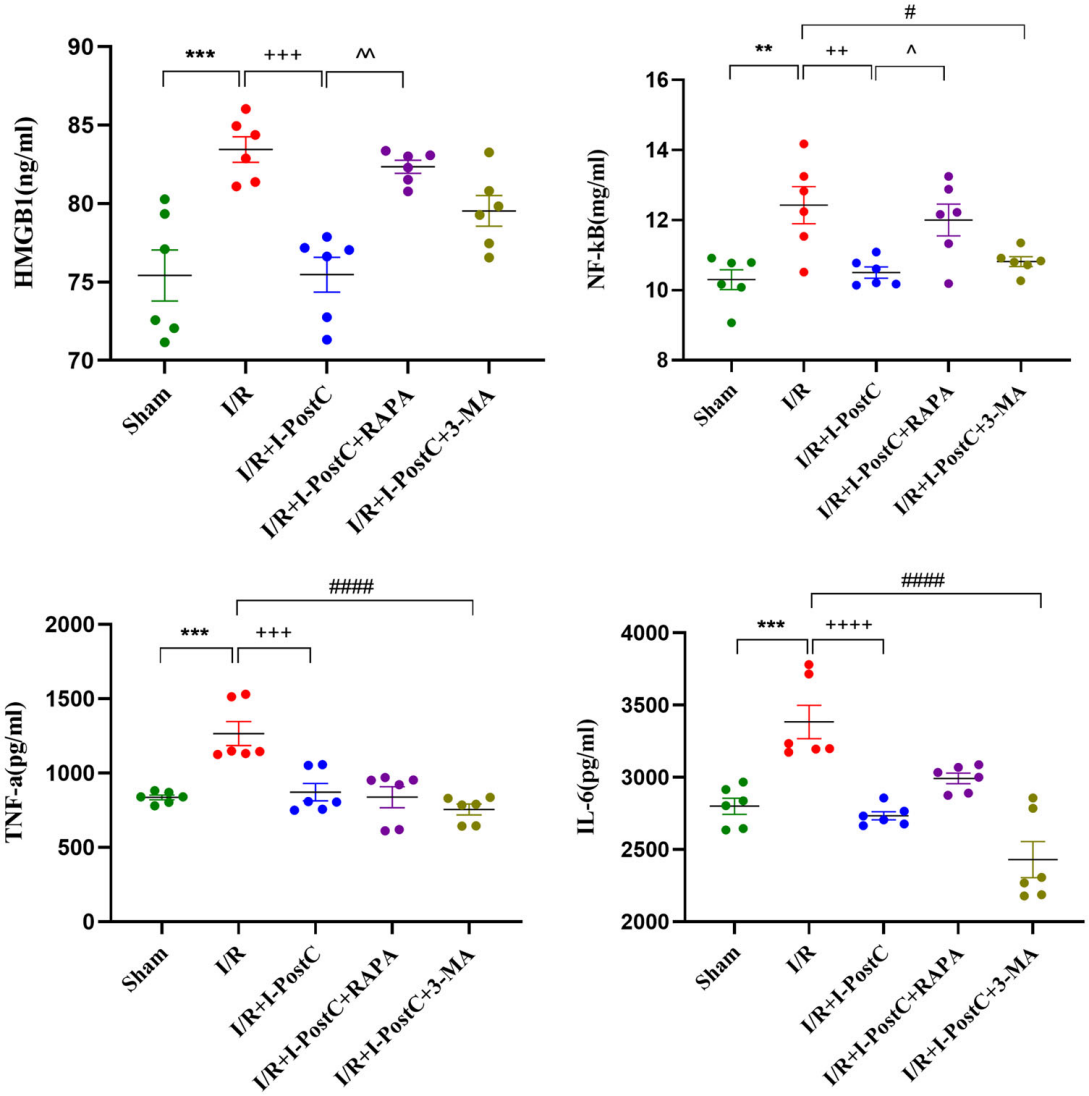
Expression levels of HMGB1, NF‑κB, TNF‑α, and IL‑6 in rat kidneys. Data are presented as the mean ± SD, *N* = 6/group. ***p<.001, **p<.01, I/R vs. sham; ^++++^*p*<.0001, ^+++^*p*<.001, ^++^*p*<.01, I/R + I-PostC vs. I/R; ^####^*p*<.0001, ^#^*p*<.05, I/R + I-PostC + 3-MA vs. I/R; ^∧∧^*p*<.01, ^∧^*p*<.05, I/R + I-PostC + RAPA vs. I/R + I-PostC. TNF‑α: tumor necrosis factor‑α; IL: interleukin.

**Figure 4.**
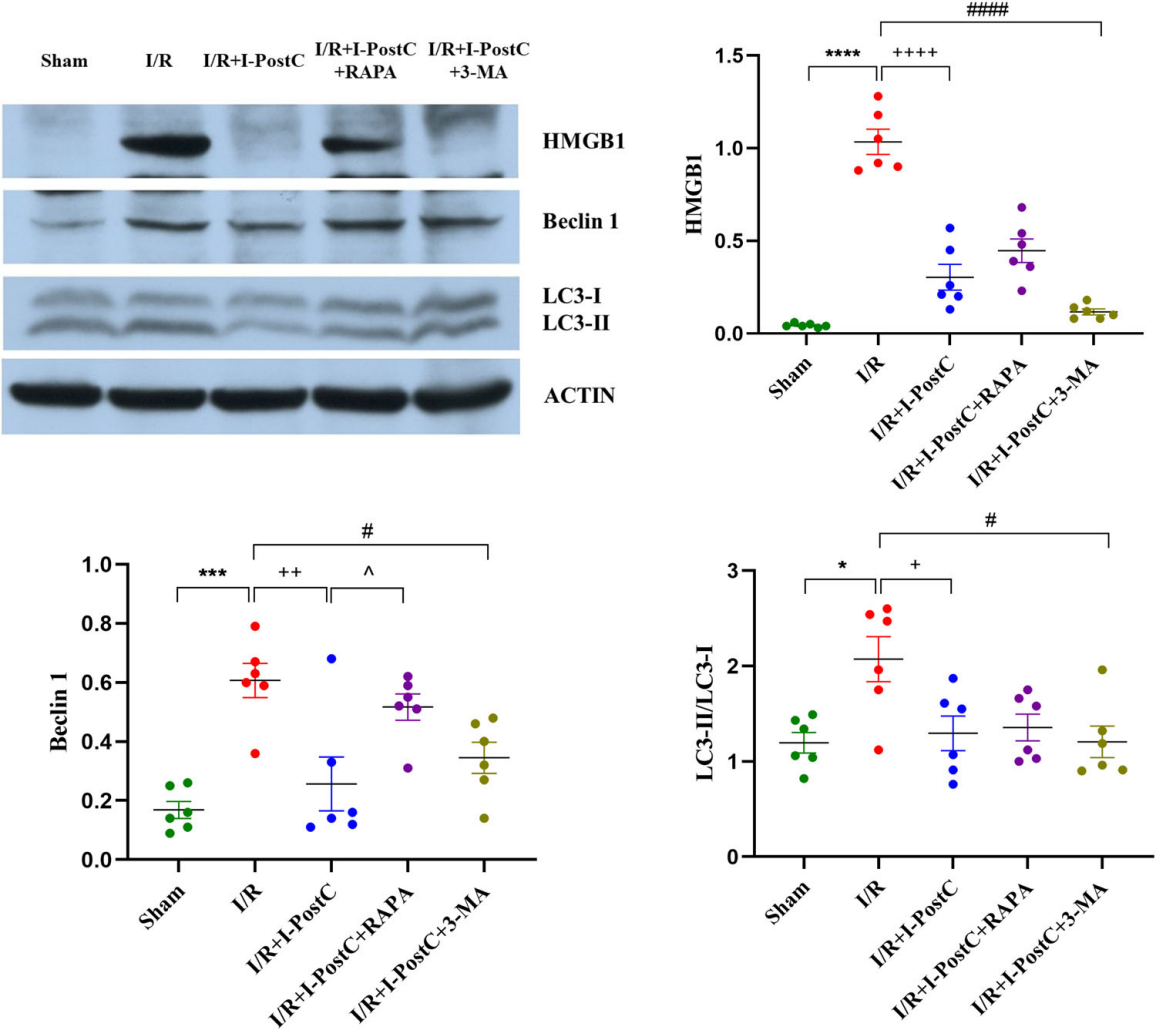
Representative western blotting images and statistical analysis of HMGB1, Beclin1 expression levels in the kidneys and LC3II/LC3I ratio in different groups. Data are presented as the mean ± SD, *N* = 6/group. ****p<.0001, ***p<.001, *p<.05, I/R vs. sham; ^++++^*p*<.0001, ^++^*p*<.01, ^+^*p*<.05, I/R + I-PostC vs. I/R; ^####^*p*<.0001, ^#^*p*<.05, I/R + I-PostC + 3-MA vs. I/R; ^∧^*p*<.05, I/R + I-PostC + RAPA vs. I/R + I-PostC.

**Figure 5.**
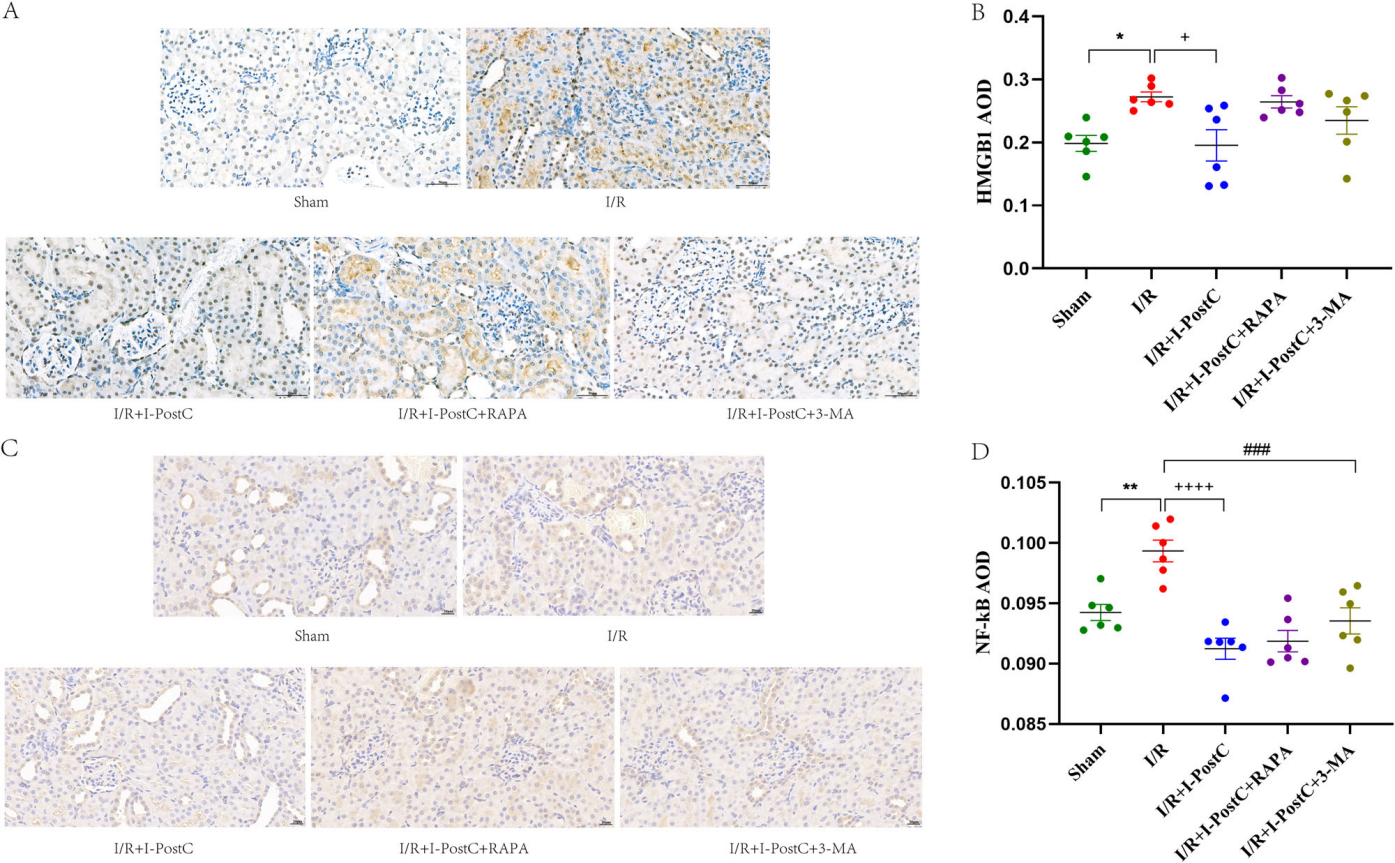
Representative immunohistochemistry images and statistical analysis of HMGB1 (A, B) and NF‑κB p65 (C, D) expression levels in rat kidney tissues. Data are presented as the mean ± SD, *N* = 6/group. **p<.01, *p<.05, I/R vs. sham; ^++++^*p*<.0001, ^+^*p*<.05, I/R + I-PostC vs. I/R; ^###^*p*<.001, I/R + I-PostC + 3-MA vs. I/R. Scale bar = 20 μm. AOD: average optical density.

Compared with the sham group, I/R significantly increased both serum and renal HMGB1 levels, as well as serum concentration of TNF-α and IL-6 (Figure 3). Semi-quantitative WB analysis showed that Beclin-1 expression level and LC3-II/LC3-I ratio were significantly higher in the I/R group than that in the sham group (Figures 4 and 5). These results demonstrated that LIR increased the levels of inflammatory markers in circulation and renal tissues, and induced autophagy activation in the kidney.

### I-PostC reduced morphological and functional kidney impairment after LIR, as well as the levels of HMGB1 and autophagy

Next, we evaluated the specific effect of I-PostC on renal injury after LIR by measuring the levels of serum inflammatory cytokines and biochemical parameters. As shown in Figures 1–3, the levels of BUN, Cr, and CK in serum were significantly decreased in the I/R + I-PostC group, and the degree of pathological injury and wet/dry weight ratio of the kidney tissues were also reduced compared to the I/R group. The levels of HMGB1 and inflammatory cytokines (TNF-α and IL-6) were also decreased in samples from the I/R + I-PostC group. Moreover, Beclin-1 expression level and LC3-II/LC3-I ratio were both significantly decreased in the I/R + I-PostC group compared to the I/R group (Figure 4). IHC showed that HMGB1 and NF-κB expression was significantly higher in the I/R group than in the sham group, while the expression levels of NF-κB in the I/R + I-PostC group were significantly decreased compared to the I/R group (Figure 5).

**Figure 6.**
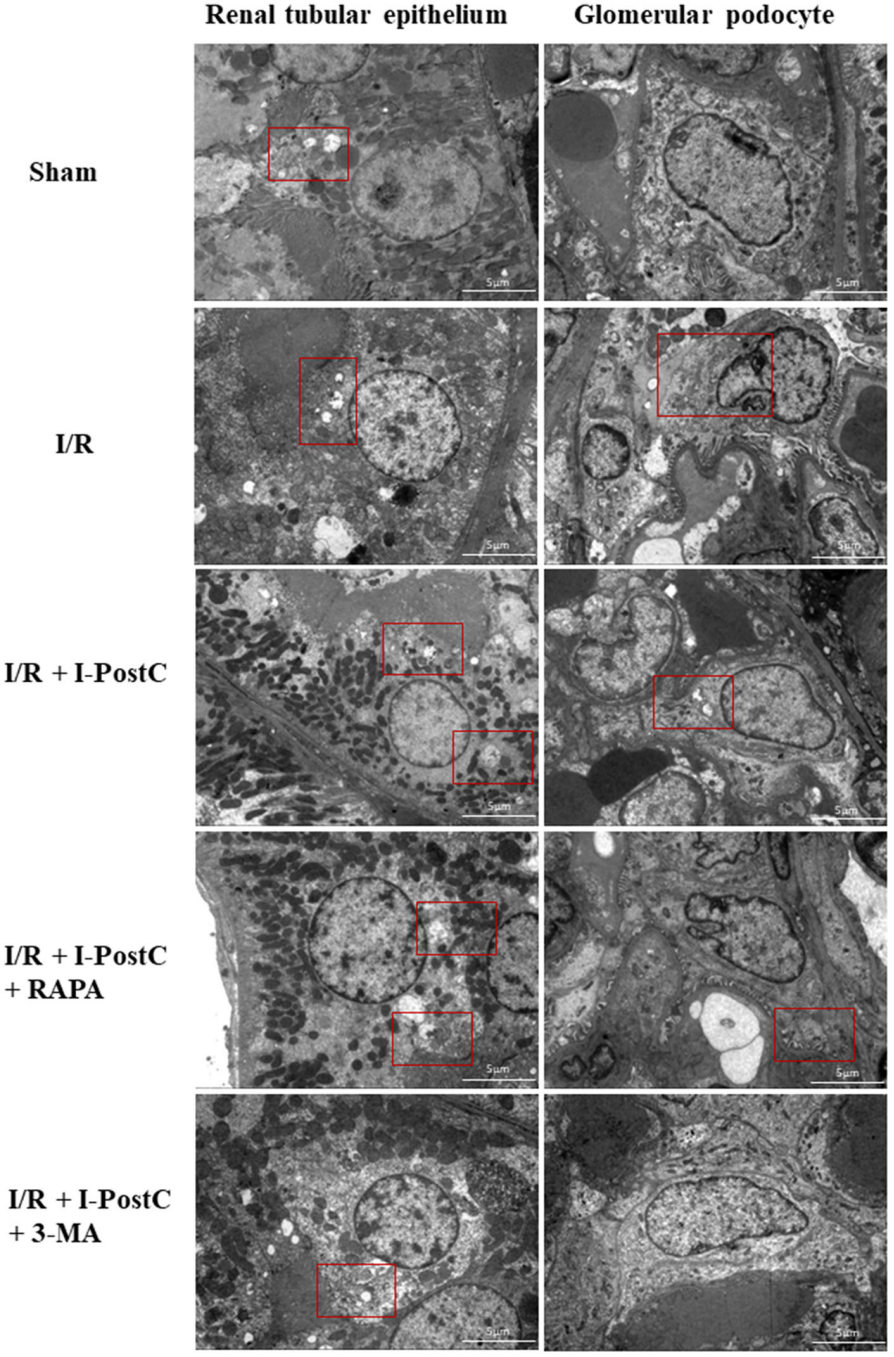
Ultrastructural changes in renal tubular epithelial cells and glomerular podocytes from rat kidneys as observed using a fluoroscopic electron microscope. Scale bar = 5 μm.

Next, we evaluated ultrastructural changes in renal tubular epithelial cells and glomerular podocytes by TEM. Normal renal tubular epithelial cells and glomerular podocytes were observed in the sham group; while in the I/R group, moderate edema of tubule epithelial cells, swelling and aggregation of organelles, glomerular podocytes showed mild to severe edema, with many glomerular foot processes fused, and a few autophagosomes were present. In the I/R + I-PostC group, mild edema of proximal tubule epithelial cells and glomerular podocytes, mitochondria showed slight swelling, and autophagosomes were isolated; extensive edema of renal tubular epithelial cells and glomerular podocytes, mitochondrial swelling, fusion of glomerular foot processes, and autophagosomes were common in the I/R + I-PostC + RAPA group. However, slight edema of renal tubular epithelial cells and glomerular podocytes, and autophagosomes were rare in the I/R + I-PostC + 3-MA group (Figure 6).

## Discussion

LIR could lead to distant organ injury, including AKI, which is often associated with rapid deterioration of renal function and high morbidity and mortality rates. Although the clinical condition has attracted global attention, the pathophysiological mechanisms of AKI after LIR remain poorly understood. The disbalance of systemic immune inflammatory response appears to play an important role in the pathogenesis of numerous diseases, and an excessive inflammatory response has been shown to be involved in the development of AKI [23]. Renal function loss due to tubular cell death is often associated with the release of DAMPs, which trigger and intensify systemic inflammation, leading to tissue damage.

DAMPs are a class of immune-stimulating molecules involved in inflammatory responses after tissue injury [10]. HMGB1 is a ubiquitously expressed non-kidney-specific DAMP that belongs to the high-mobility family of proteins and is primarily localized in the nucleus [24]. In the nucleus, HMGB1 interacts with DNA and participates in several processes, such as transcription, replication, and recombination. It has been reported that HMGB1 plays an important role in numerous acute and chronic inflammatory diseases, such as sepsis, trauma, and ischemia reperfusion injury [25]. HMGB1 could migrate to the outside of the nucleus or be released by inflammatory cells to mediate the release of downstream inflammatory factors when the above damage occurs, and was identified as a proinflammatory mediator during I/R injury [26]. Previous studies have shown that I/R could activate the body’s immune system through HMGB1-mediated signaling pathways, inducing the production of inflammatory factors and leading to cell damage [27,28]. Several groups have shown a strong association between the levels of cytokines, such as ILs, especially IL-6, or migration-inhibition factors, and the development of AKI in response to I/R. IL-6 is an important member of the cytokine network and plays a central role in acute inflammatory response [29,30]. IL-6 induces the production of C-reactive protein (CRP) and procalcitonin (PCT), which are directly related to inflammatory diseases and the severity of infection. Therefore, the inflammatory cascades induced by acute injury of intrinsic cells in the kidney are the main reason for the worsening of AKI. In addition, we previously reported that TLR4, the downstream binding molecule of HMGB1, plays a role in promoting renal injury in the acute phase [20]. A number of studies have shown that therapeutic intervention targeting HMGB1-induced inflammation can improve the survival rate in the animal models [24]. Nevertheless, few studies have identified the effect of this approach in reducing the incidence of renal inflammation and AKI after LIR.

Currently, studies on organ injury caused by LIR mainly focused on the inflammatory cascade induced by ischemia and hypoxia [5,31]. However, during the investigation of pathogenesis and treatment of AKI after I/R injury, the adaptive cellular responses, such as autophagy, have not received much attention. As an adaptive cellular response to internal and external stresses, autophagy plays an important role in the maintenance of cell homeostasis and regulation of cell survival [32]. Autophagy mediates cell survival mainly by removing damaged organelles and proteins, reducing the oxidative state of cells. In addition, autophagy plays a role in both innate and adaptive immunity [33,34].

Previous researches have confirmed that autophagy may play different roles in different renal diseases, different renal cell types, and different stages of disease progression, and the detailed regulatory mechanism and signal transmission remain to be further clarified. Recent studies have shown that autophagy is associated with the intracellular accumulation of reactive oxygen species (ROS) in response to environmental stresses, such as starvation, infection, and various diseases. During LIR, the release of a large number of oxygen free radicals leads to the accumulation of ROS in cells. As signaling molecules, ROS induce autophagy by activating c-Jun N-terminal kinase (JNK) and adenosine 5′-monophosphate (AMP)-activated protein kinase (AMPK) signal transduction pathways or by inhibiting Akt-mTOR and other pathways [35]. It was demonstrated that autophagy can phagocytose lysosomes and restore lysosomal function to prevent cell damage. Autophagy could also inhibit the activity of inflammasomes by removing damaged organelles and regulating the inflammatory response to prevent the production of inflammatory cytokines [36]. However, overactivated autophagy could induce inflammatory responses, resulting in cell and tissue damage, and even organ dysfunction [37]. It has been reported that autophagy was involved in the development of renal ischemia and reperfusion injury, since numerous experiments demonstrated the activation of autophagy during renal ischemia and reperfusion injury [33]. However, it is still under debate whether the upregulation of autophagy has a protective effect on the kidney or exacerbates kidney damage.

LC3 is a marker of autophagy, when autophagy occurs, cytoplasmic LC3 (LC3-I) will enzymically remove a small fragment of polypeptide and transform into membrane type (LC3-II). Therefore, the size of LC3-II/I ratio can estimate the level of autophagy. Beclin 1 is involved in the formation of autophagosome membrane and plays an important role in regulating apoptosis and autophagy [38]. A better understanding of the crosstalk between autophagy and the inflammatory response will contribute to the design of more effective approaches to treat AKI after LIR [39]. For example, it has been reported that exonuclear HMGB1 can directly or indirectly activate cytoplasmic Beclin1. It has been shown in tumor cells that ROS promote the transport of HMGB1 from the nucleus to the cytoplasm, while cytoplasmic HMGB1 interacts with Beclin-1, which, in turn, is released from the Beclin-1/Bcl-2 complex and induces autophagy [38,40].

In our study, the levels of HMGB1, IL-6 and TNF-α, as well as the levels of Beclin 1 and the LC3-II/LC3-I ratio, were significantly increased after LIR. Thus, LIR could promote a significant increase in the expression of HMGB1 and inflammatory factors in serum and kidney tissues, and activate autophagy in renal cells, leading to AKI. I-PostC could alleviate LIR-mediated AKI injury by downregulating the expression of HMGB1 and inflammatory cytokines in rats. Interestingly, the expression trends of inflammatory cytokines and biochemical parameters in the I/R + I-PostC + 3-MA group rats were similar to those in rats from the I-PostC group, the levels of inflammatory cytokines in circulation (IL-6) and renal tissue (NF-κB) in the I/R + I-PostC + RAPA group were higher than those in the I/R + I-PostC group. Moreover, the levels of HMGB1 and Beclin-1 in the I/R + I-PostC + RAPA group were also increased compared to the I/R + I-PostC group. These findings indicated that the expression of HMGB1 and levels of the inflammatory cytokines could be modulated with intervention in autophagy. While treatment with autophagy activator increased inflammatory cytokine expression levels and decreased renal function, suggesting that the overactivation of autophagy may play a promoting role in the process of LIR-induced AKI. Therefore, the level of autophagy significantly affects the degree of kidney injury after LIR. More importantly, I-PostC significantly reduced the levels of HMGB1, IL-6, and Beclin 1, suggesting that I-PostC could inhibit the HMGB1 expression, inflammatory cascade, autophagy activation, and regulate the interaction between tissue inflammatory response and autophagy in the renal tissue after LIR, preventing kidney damage.

Through observing the ultrastructure of renal tissue, we found that renal tubular epithelial cells exhibited obvious pathological structural changes after LIR, and NF-kB migrated and accumulated significantly in the tubular epithelium, which may suggest that LIR may lead to inflammatory response and injury of renal tubular epithelial cells, thus resulting in impaired renal function. Inhibiting autophagy could not only decrease the expression of HMGB1 and related inflammatory mediators, but also reduce the degree of renal tissue damage in microscopic and ultrastructural aspects.

Previous studies have confirmed that autophagy may regulate inflammatory response through various signaling pathways, alleviate AKI and contribute to the restoration of kidney tissue [12,15]. However, different from AKI caused by direct renal ischemia, cisplatin, urinary obstruction, and other factors, the mechanism of AKI induced by LIR in distant may be more complex. Superoxide, inflammatory reaction, hypoperfusion, myoglobin direct injury, and other factors may act and influence each other in the pathophysiological process of kidney injury. The simultaneous or sequential action of multiple injury factors causes excessive activation of autophagy and inflammatory response, resulting in renal tissue injury and function impairment. In this process, autophagy and inflammation may interact with each other, but the specific mechanism remains to be clarified. This study confirmed that I-PostC could play a protective role in the kidney by decreasing the activity of HMGB1 and inhibiting autophagy after LIR.

Our current ongoing research focuses on the cross-talk of inflammatory responses and key effectors of the autophagy pathway during the occurrence of AKI after LIR. Further understanding of the exact mechanism and the interaction between different pathways could help us find more accurate therapeutic targets and improve the prognosis.

There were several limitations in our study. First, the animal model of AKI after LIR did not perfectly mimic the clinical situation observed in patients. In reality, patients often suffer from limb ischemia due to the deformation and extrusion of the cab during car accidents or the heavy compression of building collapse. After removing the compression object, the blood flow of the limb recovers resulting in LIR. Obviously, the injury patterns encountered in the clinic are often more complex. Besides, to avoid the impact of hormone level fluctuations on the test data, the animals used in this study were all male animals, which may cause certain bias in the results. Second, the relationship between HMGB1 and inflammatory response needs to be further clarified in this injury model. Considering that HMGB1 is an important upstream molecule of the TLR4/NF-κB signaling pathway, which is related to our previous studies, further studies are needed to clarify the cross-talk mechanism of kidney inflammation and autophagy regulation after LIR. Third, only limited aspects of autophagy regulation during AKI were examined in this study. Different from AKI caused by renal ischemia, cisplatin, urinary obstruction, and other factors, the mechanism of AKI induced by limb ischemia reperfusion in distant may be more complex. Different factors may affect autophagy directly or indirectly. Our future studies will focus on related pathways that are known to regulate autophagy in AKI.

## Conclusions

In the present study, we showed that increased HMGB1 release and autophagy activation in the kidney after LIR play a vital role in AKI in rats. Our results indicated that I-PostC could protect against AKI after LIR by regulating HMGB1 expression and suppressing autophagy, providing a possible mechanism, as well as the treatment option for alleviating remote organ injury in patients with LIR. Targeting autophagy in this type of organ injury could offer therapeutic benefits.

## Data Availability

The datasets are available from the corresponding author on reasonable request.
